# Crystal structure of 5,15-bis­(4-methyl­phen­yl)-10,20-bis­(4-nitro­phen­yl)porphyrin nitro­benzene disolvate

**DOI:** 10.1107/S2056989017017868

**Published:** 2018-01-01

**Authors:** Bakhytzhan Baptayev, Salimgerey Adilov

**Affiliations:** a National Laboratory Astana, Laboratory of Chemistry, Nazarbayev University, 53 Kabanbay Batyr Ave, Astana, 010000, Kazakhstan; b Department of Chemistry, SST, Nazarbayev University, 53 Kabanbay Batyr Ave, Astana, 010000, Kazakhstan

**Keywords:** crystal structure, porphyrins, solvate, C—H⋯O hydrogen bonding

## Abstract

The whole mol­ecule of the title porphyrin, C_46_H_32_N_6_O_4_·2C_6_H_5_NO_2_, which crystallized as a nitro­benzene disolvate, is generated by inversion symmetry. The porphyrin macrocycle is almost planar, the maximum deviation from the mean plane of the non-hydrogen atoms is 0.097 (2) Å. The aryl rings at the *meso* positions are inclined to this mean plane by 74.84 (6)° for the nitro­phenyl rings and 73.37 (7)° for the tolyl rings. In the crystal, the porphyrin mol­ecules are linked by C—H⋯O hydrogen bonds, forming chains along [100]. The solvent mol­ecules are also linked by C—H⋯O hydrogen bonds, forming chains along [100]. Inter­digitation of the *p*-tolyl groups along the *c* axis creates rectangular channels in which the solvent mol­ecules are located.

## Abstract 2

The whole mol­ecule of the title porphyrin, which crystallized as a nitro­benzene disolvate, is generated by inversion symmetry. In the crystal, the porphyrin mol­ecules are linked by C—H⋯O hydrogen bonds, forming chains along [100]. The solvent mol­ecules are also linked by C—H⋯O hydrogen bonds, forming chains along [100]. Inter­digitation of the *p*-tolyl groups along the *c* axis creates rectangular channels in which the solvent mol­ecules are located.

## Chemical context   

Porphyrins and their metallated derivatives have been studied extensively for their host–guest properties (Byrn *et al.*, 1991 ▸), catalytic activity (Shultz *et al.*, 2009 ▸) and for applications in dye-sensitized solar cells (Urbani *et al.*, 2014 ▸). The presence or absence of a metal ion at the porphyrin core can greatly affect its physical properties, such as catalytic activity and crystal packing. The title compound is the free-base analogue of a previously reported zinc derivative (Adilov & Thalladi, 2007 ▸). The absence of the metal ion alters the crystal packing and these changes in the crystal structure of its nitro­benzene disolvate are discussed herein.
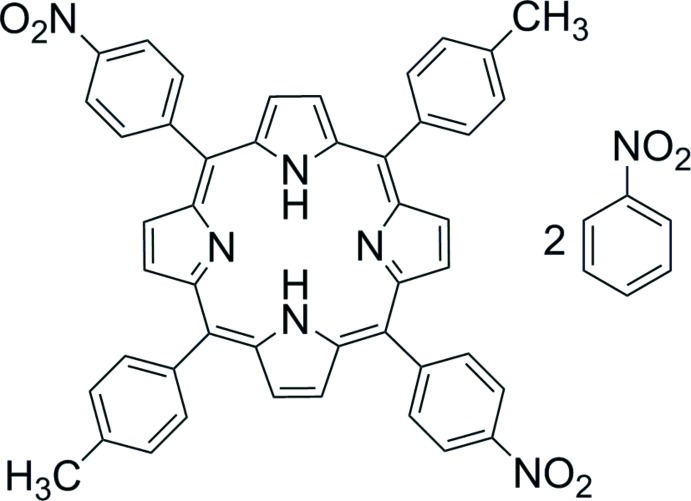


## Structural commentary   

**Figure 1:**
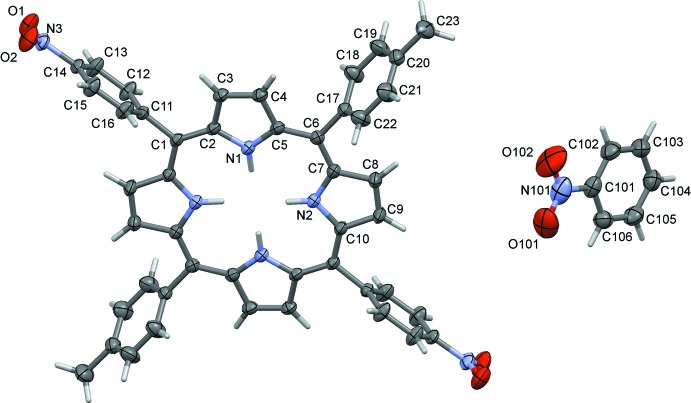
The mol­ecular structure of the title compound, with the atom labelling. Displacement ellipsoids are drawn at the 50% probability level. Unlabelled atoms are related to labelled atoms by inversion symmetry (symmetry operation: −*x*, 2 − *y*, −*z*), and only one solvent mol­ecule is shown.

The mol­ecular structure of the title compound is shown in Fig. 1 ▸. The asymmetric unit consists of half of the porphyrin mol­ecule and one nitro­benzene solvent mol­ecule. The whole mol­ecule of the porphyrin is generated by inversion symmetry. The porphyrin macrocycle is almost planar, the maximum deviation from the mean plane of the non-hydrogen atoms being 0.0970 (19) Å for atom C1 (and the symmetry-related atom). The dihedral angles between the porphyrin ring mean plane and the aryl rings at the *meso* positions are similar; 74.84 (6)° for the nitro­phenyl rings and 73.37 (7)° for the tolyl rings.

## Supra­molecular features   

**Figure 2:**
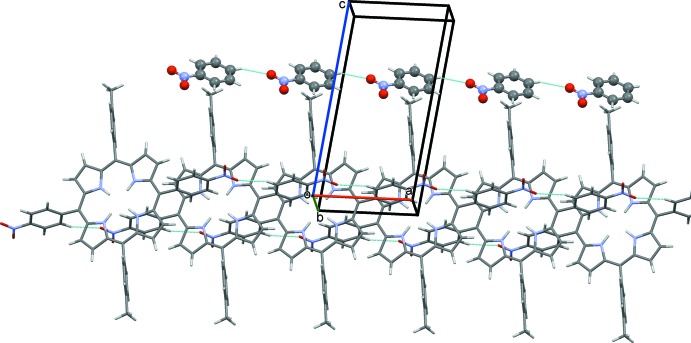
A partial view along the *b* axis of the crystal packing of the title compound. The C—H⋯O hydrogen bonds are shown as dashed lines (see Table 1 ▸).

**Figure 3:**
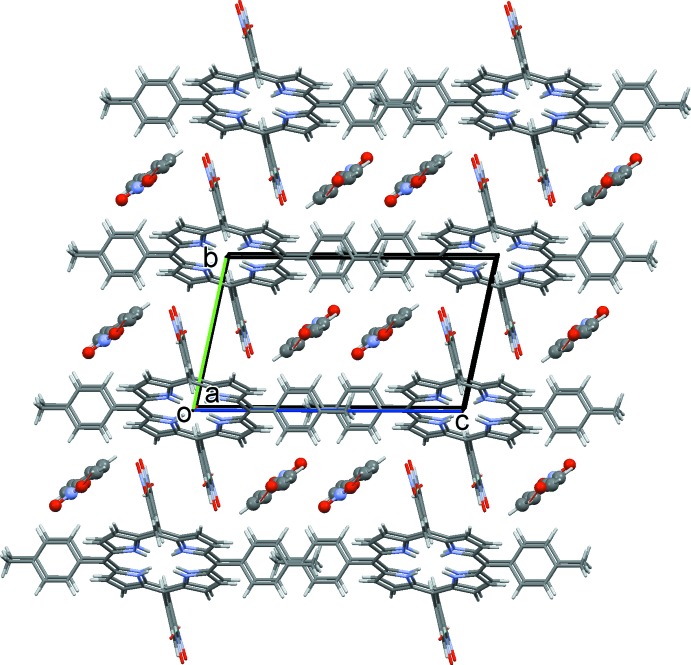
A view along the *a* axis of the inter­layer stacking in the crystal of the title compound, also showing the inter­calation of the nitro­benzene groups between the layers.

In the crystal, the solvent mol­ecules are linked by C—H⋯O hydrogen bonds [2.58 (5) Å, 129.9 (3)°] forming chains along the *a*-axis direction (Fig. 2 ▸ and Table 1 ▸). The nitro­phenyl groups of the macrocyle are projected into the inter­layer space where an oxygen of a nitro group (O2) forms a C—H⋯O hydrogen bond [2.453 (3) Å, 158.6 (2)°] with neighbouring mol­ecules, leading to the formation of chains along [100] (Fig. 2 ▸ and Table 1 ▸). Inter­digitation of the *p*-tolyl groups along the *c*-axis creates rectangular channels in which the solvent mol­ecules are located, as illustrated in Fig. 3 ▸.

## Database survey   

A search of the Cambridge Structural Database (Version 5.38, update May 2017; Groom *et al.*, 2016 ▸) for *trans* nitro­phenyl­phenyl porphyrins gave 29 hits. Apart from the zinc-metallated complex of the title compound, *catena*-[[μ_3_-5,15-bis­(*p*-tol­yl)-10,20-bis­(4-nitro­phen­yl)porphyrinato]zinc(II) nitro­benzene solvate] (CEZTUX; Adilov & Thalladi, 2007 ▸), mentioned previously, the crystal structure of the *meso*-tetra­kis­(4-nitro­phen­yl) analogue of the title compound, *viz. meso*-tetra­kis­(4-nitro­phen­yl)porphyrin nitro­benzene disolvate (BOMTEE; Seredyuk *et al.*, 2014 ▸), is of particular inter­est. While CEZTUX has the same 1:2 porphyrin-solvent ratio, it has a totally different crystal packing. Both structures, however, contain porphyrin layers and the solvent mol­ecules are inter­calated between the layers. In the title free-base, the nitro groups of the macrocycle form C—H⋯O hydrogen bonds with neighbouring mol­ecules resulting in continuous offset stacks along the *a*-axis direction. The same situation is observed in the crystal of the tetra­kis­(4-nitro­phen­yl) analogue, BOMTEE.

## Synthesis and crystallization   

The synthesis of the title compound has been described previously (Adilov & Thalladi, 2007 ▸). It crystallized as a nitro­benzene disolvate on slow evaporation of a solution in chloro­form/nitro­benzene (*v*:*v* 1:2).

## Refinement   

Crystal data, data collection and structure refinement details are summarized in Table 2 ▸. The C-bound and N-bound H atoms were included in calculated positions and refined as riding atoms: C—H = 0.95–0.98 Å, N—H = 0.88 Å with *U*_iso_(H) = 1.5*U*_eq_(C-meth­yl) and 1.2*U*_eq_(N, C) for other H atoms. The two NH H atoms in the porphyrin core are disordered over the four pyrrole N-atoms, and were refined with occupancies of 0.5 each.

## Supplementary Material

Crystal structure: contains datablock(s) I, Global. DOI: 10.1107/S2056989017017868/su5408sup1.cif

Structure factors: contains datablock(s) I. DOI: 10.1107/S2056989017017868/su5408Isup2.hkl

CCDC reference: 1811082

Additional supporting information:  crystallographic information; 3D view; checkCIF report

## Figures and Tables

**Table 1:** Hydrogen-bond geometry (Å, °)

*D*—H⋯*A*	*D*—H	H⋯*A*	*D*⋯*A*	*D*—H⋯*A*
C12—H12⋯O2^i^	0.95 (1)	2.45 (1)	3.355 (3)	159 (1)
C104—H104⋯O102^i^	0.95 (1)	2.58 (1)	3.272 (4)	130 (1)

Symmetry code: (i) 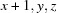.

**Table 2:** Experimental details

Crystal data	
Chemical formula	C_46_H_32_N_6_O_4_·2C_6_H_5_NO_2_
*M*_r_	979.03
Crystal system, space group	Triclinic, *P* 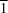
Temperature (K)	193
*a*, *b*, *c* (Å)	7.957 (3), 9.656 (3), 16.568 (5)
α, β, γ (°)	76.710 (5), 79.440 (5), 78.173 (5)
*V* (Å^3^)	1200.1 (7)
*Z*	1
Radiation type	Mo *K*α
μ (mm^−1^)	0.09
Crystal size (mm)	0.2 × 0.15 × 0.1

Data collection	
Diffractometer	Bruker SMART CCD area detector
Absorption correction	Multi-scan (*SADABS*; Bruker, 2005 ▸)
*T*_min_, *T*_max_	0.830, 0.991
No. of measured, independent and observed [*I* > 2σ(*I*)] reflections	7891, 5512, 4072
*R*_int_	0.066
(sin θ/λ)_max_ (Å^−1^)	0.658

Refinement	
*R*[*F* ^2^ > 2σ(*F* ^2^)], *wR*(*F* ^2^), *S*	0.064, 0.189, 1.05
No. of reflections	5512
No. of parameters	334
H-atom treatment	All H-atom parameters refined
Δρ_max_, Δρ_min_ (e Å^−3^)	0.80, −0.37

Computer programs: *SMART* and *SAINT* (Bruker, 2005 ▸), *SIR2004* (Burla *et al.*, 2007 ▸), *Olex2.refine* (Bourhis *et al.*, 2015 ▸), *OLEX2* (Dolomanov *et al.*, 2009 ▸), *Mercury* (Macrae *et al.*, 2008 ▸), *SHELXTL* (Sheldrick, 2008 ▸) and *publCIF* (Westrip, 2010 ▸).

## supplementary crystallographic information

### Crystal data

**Table:**

C_46_H_32_N_6_O_4_·2C_6_H_5_NO_2_	*Z* = 1
*M**_r_* = 979.03	*F*(000) = 510.2496
Triclinic, *P*1	*D*_x_ = 1.355 Mg m^−^^3^
*a* = 7.957 (3) Å	Mo *K*α radiation, λ = 0.71073 Å
*b* = 9.656 (3) Å	Cell parameters from 832 reflections
*c* = 16.568 (5) Å	θ = 5.5–55.4°
α = 76.710 (5)°	µ = 0.09 mm^−^^1^
β = 79.440 (5)°	*T* = 193 K
γ = 78.173 (5)°	Plate, dark red
*V* = 1200.1 (7) Å^3^	0.2 × 0.15 × 0.1 mm

### Data collection

**Table:**

Bruker SMART CCD area detector diffractometer	5512 independent reflections
Radiation source: fine-focus sealed tube	4072 reflections with *I* > 2σ(*I*)
Graphite monochromator	*R*_int_ = 0.066
φ and ω scans	θ_max_ = 27.9°, θ_min_ = 1.3°
Absorption correction: multi-scan (SADABS; Bruker, 2005)	*h* = −10→10
*T*_min_ = 0.830, *T*_max_ = 0.991	*k* = −12→12
7891 measured reflections	*l* = −14→21

### Refinement

**Table:**

Refinement on *F*^2^	42 constraints
Least-squares matrix: full	Primary atom site location: structure-invariant direct methods
*R*[*F*^2^ > 2σ(*F*^2^)] = 0.064	All H-atom parameters refined
*wR*(*F*^2^) = 0.189	*w* = 1/[σ^2^(*F*_o_^2^) + (0.0885*P*)^2^ + 0.7087*P*] where *P* = (*F*_o_^2^ + 2*F*_c_^2^)/3
*S* = 1.05	(Δ/σ)_max_ = 0.001
5512 reflections	Δρ_max_ = 0.80 e Å^−^^3^
334 parameters	Δρ_min_ = −0.37 e Å^−^^3^
0 restraints	

### Fractional atomic coordinates and isotropic or equivalent isotropic displacement parameters (Å^2^)

**Table:**

	*x*	*y*	*z*	*U*_iso_*/*U*_eq_	Occ. (<1)
C1	−0.4023 (2)	1.1991 (2)	−0.03569 (12)	0.0242 (4)	
N1	−0.2172 (2)	1.08626 (18)	0.07401 (10)	0.0253 (4)	
H1	−0.1213 (2)	1.05861 (18)	0.04157 (10)	0.0304 (4)*	0.500000
O1	−1.0421 (2)	1.6738 (2)	−0.15516 (13)	0.0524 (5)	
C2	−0.3711 (2)	1.1584 (2)	0.04770 (13)	0.0254 (4)	
N2	0.1210 (2)	0.91439 (18)	0.10379 (10)	0.0249 (4)	
H2	0.0670 (2)	0.94670 (18)	0.05953 (10)	0.0299 (4)*	0.500000
O2	−1.1746 (2)	1.4941 (2)	−0.09963 (14)	0.0598 (6)	
C3	−0.4958 (3)	1.1826 (2)	0.11990 (13)	0.0293 (4)	
H3	−0.6126 (3)	1.2307 (2)	0.11960 (13)	0.0351 (5)*	
N3	−1.0444 (2)	1.5482 (2)	−0.11856 (12)	0.0348 (4)	
C4	−0.4157 (3)	1.1240 (2)	0.18851 (13)	0.0294 (4)	
H4	−0.4667 (3)	1.1223 (2)	0.24519 (13)	0.0353 (5)*	
C5	−0.2391 (3)	1.0648 (2)	0.15975 (13)	0.0257 (4)	
C6	−0.1130 (3)	0.9975 (2)	0.21153 (12)	0.0258 (4)	
C7	0.0548 (3)	0.9304 (2)	0.18412 (12)	0.0263 (4)	
C8	0.1855 (3)	0.8648 (3)	0.23783 (13)	0.0331 (5)	
H8	0.1742 (3)	0.8620 (3)	0.29623 (13)	0.0397 (6)*	
C9	0.3270 (3)	0.8079 (3)	0.18946 (13)	0.0328 (5)	
H9	0.4332 (3)	0.7571 (3)	0.20771 (13)	0.0394 (6)*	
C10	0.2867 (2)	0.8387 (2)	0.10502 (12)	0.0253 (4)	
C11	−0.5730 (2)	1.2911 (2)	−0.05372 (12)	0.0245 (4)	
C12	−0.5801 (3)	1.4362 (2)	−0.08993 (16)	0.0356 (5)	
H12	−0.4777 (3)	1.4775 (2)	−0.10072 (16)	0.0427 (6)*	
C13	−0.7349 (3)	1.5219 (2)	−0.11066 (15)	0.0360 (5)	
H13	−0.7399 (3)	1.6216 (2)	−0.13496 (15)	0.0432 (6)*	
C14	−0.8809 (2)	1.4592 (2)	−0.09523 (13)	0.0274 (4)	
C15	−0.8795 (3)	1.3162 (3)	−0.05707 (17)	0.0395 (6)	
H15	−0.9830 (3)	1.2761 (3)	−0.04532 (17)	0.0474 (7)*	
C16	−0.7239 (3)	1.2319 (2)	−0.03617 (16)	0.0370 (5)	
H16	−0.7207 (3)	1.1332 (2)	−0.00974 (16)	0.0443 (6)*	
C17	−0.1603 (3)	0.9982 (2)	0.30326 (12)	0.0274 (4)	
C18	−0.1736 (4)	1.1229 (3)	0.33314 (15)	0.0439 (6)	
H18	−0.1544 (4)	1.2097 (3)	0.29475 (15)	0.0526 (7)*	
C19	−0.2144 (4)	1.1238 (3)	0.41821 (16)	0.0493 (7)	
H19	−0.2223 (4)	1.2108 (3)	0.43721 (16)	0.0591 (8)*	
C20	−0.2439 (3)	0.9990 (3)	0.47565 (14)	0.0382 (5)	
C21	−0.2353 (4)	0.8755 (3)	0.44615 (15)	0.0463 (6)	
H21	−0.2577 (4)	0.7895 (3)	0.48459 (15)	0.0556 (7)*	
C22	−0.1943 (3)	0.8742 (3)	0.36067 (14)	0.0414 (6)	
H22	−0.1895 (3)	0.7877 (3)	0.34163 (14)	0.0497 (7)*	
C23	−0.2861 (4)	0.9995 (4)	0.56844 (16)	0.0555 (8)	
H23a	−0.1805 (7)	0.963 (2)	0.5947 (3)	0.0833 (12)*	
H23b	−0.332 (3)	1.0984 (5)	0.57653 (16)	0.0833 (12)*	
H23c	−0.373 (2)	0.9378 (19)	0.5942 (3)	0.0833 (12)*	
C101	0.6144 (3)	0.4878 (3)	0.62860 (17)	0.0449 (6)	
N101	0.4624 (4)	0.4425 (3)	0.6107 (2)	0.0671 (8)	
O101	0.4858 (4)	0.3604 (3)	0.5613 (2)	0.0894 (9)	
C102	0.5908 (4)	0.5772 (3)	0.68566 (18)	0.0520 (7)	
H102	0.4780 (4)	0.6110 (3)	0.71179 (18)	0.0624 (8)*	
O102	0.3212 (3)	0.4896 (4)	0.6447 (3)	0.1102 (11)	
C103	0.7342 (4)	0.6154 (4)	0.70340 (19)	0.0574 (8)	
H103	0.7214 (4)	0.6759 (4)	0.74271 (19)	0.0689 (9)*	
C104	0.8975 (4)	0.5666 (3)	0.66456 (19)	0.0528 (7)	
H104	0.9964 (4)	0.5943 (3)	0.67696 (19)	0.0633 (8)*	
C105	0.9175 (4)	0.4789 (3)	0.60842 (18)	0.0504 (7)	
H105	1.0305 (4)	0.4455 (3)	0.58236 (18)	0.0605 (8)*	
C106	0.7757 (4)	0.4377 (3)	0.58887 (17)	0.0472 (6)	
H106	0.7891 (4)	0.3771 (3)	0.54953 (17)	0.0566 (7)*	

### Atomic displacement parameters (Å^2^)

**Table:**

	*U*^11^	*U*^22^	*U*^33^	*U*^12^	*U*^13^	*U*^23^
C1	0.0182 (9)	0.0227 (9)	0.0314 (10)	−0.0003 (7)	−0.0061 (7)	−0.0052 (8)
N1	0.0216 (8)	0.0256 (8)	0.0279 (9)	0.0008 (6)	−0.0059 (6)	−0.0060 (7)
O1	0.0374 (10)	0.0453 (11)	0.0675 (13)	0.0024 (8)	−0.0168 (9)	0.0018 (9)
C2	0.0206 (9)	0.0225 (9)	0.0323 (10)	0.0002 (7)	−0.0044 (8)	−0.0066 (8)
N2	0.0204 (8)	0.0260 (8)	0.0276 (8)	0.0002 (6)	−0.0061 (6)	−0.0054 (7)
O2	0.0261 (9)	0.0670 (14)	0.0843 (15)	−0.0090 (9)	−0.0175 (9)	−0.0028 (11)
C3	0.0224 (9)	0.0308 (11)	0.0326 (11)	0.0015 (8)	−0.0029 (8)	−0.0084 (8)
N3	0.0203 (9)	0.0442 (12)	0.0388 (10)	−0.0004 (8)	−0.0083 (7)	−0.0074 (9)
C4	0.0243 (10)	0.0304 (11)	0.0315 (11)	0.0002 (8)	−0.0015 (8)	−0.0084 (8)
C5	0.0242 (9)	0.0230 (9)	0.0294 (10)	−0.0018 (7)	−0.0035 (8)	−0.0063 (8)
C6	0.0253 (10)	0.0247 (10)	0.0273 (10)	−0.0020 (8)	−0.0043 (8)	−0.0063 (8)
C7	0.0251 (10)	0.0261 (10)	0.0272 (10)	−0.0004 (8)	−0.0067 (8)	−0.0059 (8)
C8	0.0304 (11)	0.0401 (12)	0.0271 (10)	0.0026 (9)	−0.0099 (8)	−0.0064 (9)
C9	0.0254 (10)	0.0397 (12)	0.0309 (11)	0.0040 (9)	−0.0106 (8)	−0.0054 (9)
C10	0.0211 (9)	0.0249 (10)	0.0298 (10)	−0.0009 (7)	−0.0072 (8)	−0.0047 (8)
C11	0.0185 (9)	0.0260 (10)	0.0289 (10)	−0.0008 (7)	−0.0045 (7)	−0.0070 (8)
C12	0.0185 (9)	0.0302 (11)	0.0546 (14)	−0.0057 (8)	−0.0073 (9)	0.0015 (10)
C13	0.0237 (10)	0.0265 (11)	0.0529 (14)	−0.0019 (8)	−0.0103 (9)	0.0034 (10)
C14	0.0173 (9)	0.0333 (11)	0.0309 (10)	0.0008 (8)	−0.0060 (7)	−0.0073 (8)
C15	0.0232 (10)	0.0350 (12)	0.0637 (16)	−0.0092 (9)	−0.0140 (10)	−0.0068 (11)
C16	0.0282 (11)	0.0241 (11)	0.0595 (15)	−0.0053 (9)	−0.0127 (10)	−0.0044 (10)
C17	0.0240 (9)	0.0308 (10)	0.0259 (10)	0.0011 (8)	−0.0052 (8)	−0.0068 (8)
C18	0.0649 (17)	0.0350 (13)	0.0320 (12)	−0.0109 (12)	−0.0054 (11)	−0.0064 (10)
C19	0.0735 (19)	0.0401 (14)	0.0374 (13)	−0.0074 (13)	−0.0073 (12)	−0.0167 (11)
C20	0.0360 (12)	0.0456 (14)	0.0303 (11)	0.0030 (10)	−0.0069 (9)	−0.0092 (10)
C21	0.0621 (17)	0.0396 (14)	0.0324 (12)	−0.0089 (12)	−0.0013 (11)	−0.0019 (10)
C22	0.0579 (15)	0.0334 (12)	0.0321 (12)	−0.0090 (11)	−0.0008 (10)	−0.0082 (9)
C23	0.0658 (18)	0.0635 (19)	0.0312 (13)	0.0071 (15)	−0.0060 (12)	−0.0139 (12)
C101	0.0386 (13)	0.0421 (14)	0.0511 (15)	−0.0067 (11)	−0.0121 (11)	0.0008 (11)
N101	0.0556 (17)	0.0549 (16)	0.092 (2)	−0.0197 (13)	−0.0255 (15)	0.0042 (15)
O101	0.103 (2)	0.0722 (17)	0.111 (2)	−0.0413 (15)	−0.0389 (17)	−0.0134 (16)
C102	0.0391 (14)	0.0584 (17)	0.0512 (16)	0.0049 (12)	−0.0012 (12)	−0.0121 (13)
O102	0.0402 (14)	0.114 (3)	0.179 (3)	−0.0187 (15)	−0.0151 (16)	−0.029 (2)
C103	0.0534 (17)	0.0654 (19)	0.0557 (17)	0.0089 (14)	−0.0165 (13)	−0.0266 (15)
C104	0.0424 (14)	0.0585 (17)	0.0599 (17)	−0.0046 (13)	−0.0170 (12)	−0.0125 (14)
C105	0.0374 (13)	0.0565 (17)	0.0517 (15)	−0.0014 (12)	−0.0001 (11)	−0.0103 (13)
C106	0.0528 (15)	0.0420 (14)	0.0444 (14)	−0.0029 (12)	−0.0046 (12)	−0.0103 (11)

### Geometric parameters (Å, º)

**Table:**

C1—C2	1.400 (3)	C15—H15	0.9500
C1—C10^i^	1.403 (3)	C15—C16	1.390 (3)
C1—C11	1.501 (2)	C16—H16	0.9500
N1—H1	0.8800	C17—C18	1.382 (3)
N1—C2	1.372 (2)	C17—C22	1.388 (3)
N1—C5	1.371 (3)	C18—H18	0.9500
O1—N3	1.226 (3)	C18—C19	1.389 (3)
C2—C3	1.438 (3)	C19—H19	0.9500
N2—H2	0.8800	C19—C20	1.386 (4)
N2—C7	1.370 (3)	C20—C21	1.374 (4)
N2—C10	1.372 (2)	C20—C23	1.513 (3)
O2—N3	1.213 (3)	C21—H21	0.9500
C3—H3	0.9500	C21—C22	1.396 (3)
C3—C4	1.357 (3)	C22—H22	0.9500
N3—C14	1.468 (2)	C23—H23a	0.9800
C4—H4	0.9500	C23—H23b	0.9800
C4—C5	1.442 (3)	C23—H23c	0.9800
C5—C6	1.399 (3)	C101—N101	1.466 (4)
C6—C7	1.402 (3)	C101—C102	1.385 (4)
C6—C17	1.498 (3)	C101—C106	1.378 (4)
C7—C8	1.444 (3)	N101—O101	1.231 (4)
C8—H8	0.9500	N101—O102	1.210 (4)
C8—C9	1.352 (3)	C102—H102	0.9500
C9—H9	0.9500	C102—C103	1.368 (4)
C9—C10	1.443 (3)	C103—H103	0.9500
C11—C12	1.385 (3)	C103—C104	1.381 (4)
C11—C16	1.389 (3)	C104—H104	0.9500
C12—H12	0.9500	C104—C105	1.364 (4)
C12—C13	1.388 (3)	C105—H105	0.9500
C13—H13	0.9500	C105—C106	1.382 (4)
C13—C14	1.374 (3)	C106—H106	0.9500
C14—C15	1.379 (3)		
			
C10^i^—C1—C2	125.44 (17)	H15—C15—C14	120.65 (12)
C11—C1—C2	118.19 (17)	C16—C15—C14	118.7 (2)
C11—C1—C10^i^	116.37 (17)	C16—C15—H15	120.65 (13)
C2—N1—H1	126.02 (11)	C15—C16—C11	120.4 (2)
C5—N1—H1	126.02 (10)	H16—C16—C11	119.82 (12)
C5—N1—C2	107.95 (16)	H16—C16—C15	119.82 (13)
N1—C2—C1	125.50 (18)	C18—C17—C6	121.0 (2)
C3—C2—C1	125.69 (17)	C22—C17—C6	121.06 (19)
C3—C2—N1	108.79 (17)	C22—C17—C18	118.0 (2)
C7—N2—H2	126.28 (10)	H18—C18—C17	119.36 (14)
C10—N2—H2	126.28 (11)	C19—C18—C17	121.3 (2)
C10—N2—C7	107.43 (16)	C19—C18—H18	119.36 (15)
H3—C3—C2	126.37 (11)	H19—C19—C18	119.68 (15)
C4—C3—C2	107.25 (18)	C20—C19—C18	120.6 (2)
C4—C3—H3	126.37 (12)	C20—C19—H19	119.68 (14)
O2—N3—O1	123.68 (19)	C21—C20—C19	118.4 (2)
C14—N3—O1	118.12 (18)	C23—C20—C19	120.6 (2)
C14—N3—O2	118.2 (2)	C23—C20—C21	121.0 (2)
H4—C4—C3	126.23 (12)	H21—C21—C20	119.43 (15)
C5—C4—C3	107.55 (18)	C22—C21—C20	121.1 (2)
C5—C4—H4	126.23 (12)	C22—C21—H21	119.43 (15)
C4—C5—N1	108.44 (17)	C21—C22—C17	120.6 (2)
C6—C5—N1	126.46 (18)	H22—C22—C17	119.72 (13)
C6—C5—C4	125.10 (19)	H22—C22—C21	119.72 (15)
C7—C6—C5	125.03 (19)	H23a—C23—C20	109.5
C17—C6—C5	117.50 (17)	H23b—C23—C20	109.5
C17—C6—C7	117.47 (17)	H23b—C23—H23a	109.5
C6—C7—N2	126.41 (17)	H23c—C23—C20	109.5
C8—C7—N2	109.04 (17)	H23c—C23—H23a	109.5
C8—C7—C6	124.54 (19)	H23c—C23—H23b	109.5
H8—C8—C7	126.39 (12)	C102—C101—N101	118.9 (3)
C9—C8—C7	107.22 (18)	C106—C101—N101	118.6 (3)
C9—C8—H8	126.39 (12)	C106—C101—C102	122.5 (3)
H9—C9—C8	126.33 (12)	O101—N101—C101	118.2 (3)
C10—C9—C8	107.34 (18)	O102—N101—C101	117.9 (3)
C10—C9—H9	126.33 (11)	O102—N101—O101	123.9 (3)
N2—C10—C1^i^	126.24 (18)	H102—C102—C101	120.88 (16)
C9—C10—C1^i^	124.76 (17)	C103—C102—C101	118.2 (2)
C9—C10—N2	108.96 (17)	C103—C102—H102	120.88 (17)
C12—C11—C1	119.81 (17)	H103—C103—C102	119.77 (17)
C16—C11—C1	120.80 (18)	C104—C103—C102	120.5 (3)
C16—C11—C12	119.37 (18)	C104—C103—H103	119.77 (18)
H12—C12—C11	119.57 (11)	H104—C104—C103	119.89 (18)
C13—C12—C11	120.86 (19)	C105—C104—C103	120.2 (3)
C13—C12—H12	119.57 (13)	C105—C104—H104	119.89 (17)
H13—C13—C12	120.77 (13)	H105—C105—C104	119.47 (17)
C14—C13—C12	118.5 (2)	C106—C105—C104	121.1 (3)
C14—C13—H13	120.77 (12)	C106—C105—H105	119.47 (16)
C13—C14—N3	118.88 (19)	C105—C106—C101	117.5 (3)
C15—C14—N3	118.92 (18)	H106—C106—C101	121.26 (17)
C15—C14—C13	122.19 (18)	H106—C106—C105	121.26 (16)

Symmetry code: (i) −*x*, −*y*+2, −*z*.

### Hydrogen-bond geometry (Å, º)

**Table:**

*D*—H···*A*	*D*—H	H···*A*	*D*···*A*	*D*—H···*A*
C12—H12···O2^ii^	0.95 (1)	2.45 (1)	3.355 (3)	159 (1)
C104—H104···O102^ii^	0.95 (1)	2.58 (1)	3.272 (4)	130 (1)

Symmetry code: (ii) *x*+1, *y*, *z*.
